# Rehabilitation utilization of non-migrant and migrant persons with back pain: A cohort study using different definitions of migrant background

**DOI:** 10.1016/j.eclinm.2022.101351

**Published:** 2022-03-21

**Authors:** David Fauser, Hannes Banaschak, Julia-Marie Zimmer, André Golla, Nadine Schmitt, Wilfried Mau, Matthias Bethge

**Affiliations:** a Institute for Social Medicine and Epidemiology, University of Lübeck, Lübeck, Germany; b Institute for Rehabilitation Medicine, Interdisciplinary Centre of Health Sciences, Medical Faculty, Martin Luther University Halle-Wittenberg, Halle (Saale), Germany

**Keywords:** Medical rehabilitation, Migration background, Assessment, Utilization

## Abstract

**Background:**

Medical rehabilitation (MR) by the German Pension Insurance is approved to maintain and to restore work ability and to avoid disability pensions. Studies on the rehabilitation utilization by people with a migration background (PMB) compared to people without a migration background (non-PMB) showed heterogeneous results, which may be partly due to different definitions of migration status. The aim of this paper was to test whether there are differences in utilization of MR between employed PMB and non-PMB with self-reported back pain.

**Methods:**

We used data from a large German cohort study that analyzed the effectiveness of MR for individuals with back pain and was conducted between 1st January 2017 and 31st December 2019. Employees aged 45 to 59 years who reported back pain in the last three months completed the baseline questionnaire in 2017. We used four definitions of migration background (MB) to differentiate by first- and second-generation migration, by one- and two-sided migration background, by language, or by nationality. Data on rehabilitation utilization was extracted from administrative records covering the period until the end of 2018.

**Findings:**

Data of 6,713 participants were included, and 514 individuals utilized MR during follow-up. Adjusted analyses showed a decreased risk of rehabilitation utilization in people with a first-generation MB (HR = 0·46; 95% CI 0·29; 0·72), people with a two-sided MB (HR = 0·47; 95% CI 0·31; 0·72), people whose native language was not German (HR = 0·52; 95% CI 0·30; 0·91), and people without German nationality (HR = 0·29; 95% CI 0·12; 0·72) when compared to non-PMB.

**Interpretation:**

This study showed that employees with a MB reporting back pain had a significantly reduced risk for utilization of rehabilitation services. This underutilization could be observed considering different definitions of MB. Future research on rehabilitation utilization by PMB should consider the impact of different definitions on the results.

**Funding:**

The study was funded by the 10.13039/501100001659German Research Foundation (grant numbers: BE 5885/2–1; MA 6981/2–1). The German Research Foundation functions as a self-governing institution for the promotion of science and research in Germany.

**Research in context**

**Evidence before this study**

We searched PubMed with no language restriction for studies analyzing the utilization of rehabilitation in person with a migration background in Germany from January 1, 2000 to October 31, 2021. We used search terms ("rehabilitation") AND ("migration background" OR "migrant background") AND ("Germany"). The studies identified, including a scoping review from 2020, captured migration background differently (e.g., via nationality) and showed ambiguous results of rehabilitation utilization in persons with a migration background.

**Added value of this study**

Our study uses a large sample of individuals with back pain and showed that individuals with migration biographies are less likely to apply for and use rehabilitation, even when adjusting for family and workplace barriers in addition to health and socioeconomic characteristics. Moreover, the results showed that underutilization of medical rehabilitation in persons with a migration background exists regardless of how migrant background is assessed. However, the extent of underutilization depends in part on how the construct of migration background is understood and operationalized.

**Implications of all the available evidence**

For individuals with back pain, those with a history of migration are less likely to use medical rehabilitation, given the same socioeconomic and health conditions. As medical rehabilitation is an important service for people with chronic conditions in Germany to achieve equal participation in working life and to prevent age-related poverty by building up pension entitlements, equal access for migrants with health problems is essential to avoid a double disadvantage.

Alt-text: Unlabelled box

## Introduction

The Federal Republic of Germany has been a country of immigration since its founding. According to the Federal Statistical Office, about 21 million people with a migration background (PMB), i.e. people who themselves or at least one of whose parents does not have German citizenship by birth, lived in Germany in 2019.^1^ The proportion of PMB in the working-age population between 15 and 65 years of age corresponds to about 27·6% and will continue to increase.^1^^,^^2^ As in other industrial countries, musculoskeletal disorders are one of the main causes of absenteeism, incapacity to work, and early retirement in Germany.^3^ Back pain in the German population is the most common musculoskeletal disease, with a one-year prevalence of 61%, of which about one-third have severe back pain.^4^ Sick leave, disability, and health care utilization due to back pain present high economic burdens for the German health care system and others worldwide.^5^^,^^6^ Data on the prevalence of back pain in the migrant population in Germany are not available. However, PMB are similarly likely to report suffering from a chronic disease as people without a migration background (non-PMB).^7^ Due to a worse social situation and poorer working conditions when compared to non-PMB, they also suffer more frequently from occupational diseases.^8^

In order to maintain and restore work ability and to avoid disability pensions, the German Pension Insurance funds medical rehabilitation services (MR).^9^ In addition to German citizens, all persons with a secure residency status in Germany have formal legal access to MR. Participation in a rehabilitation program either requires a claim by the person in need or may be initiated directly by the hospital if treatment in a hospital was needed (e.g., spinal fusion). Rehabilitation programs are usually provided as inpatient or outpatient programs lasting three to four weeks with a treatment dose of about 60 h. The program is delivered by a multi-professional team, and contains mainly exercise, social counseling, patient education, and psychological group sessions.

Studies on the utilization of MR by PMB in Germany paint a heterogeneous picture when compared to non-PMB.^10^ An analysis of routine data from the German Pension Insurance showed a lower utilization of MR before a disability pension by foreign nationals when compared to that by Germans.^11^ Further studies based on administrative data from the German Pension Insurance and Health Insurance also showed lower utilization by non-German insured individuals.^12^ Similar results were obtained from analyses using data from a large representative study, which indicated that foreign nationals were less likely to use MR than Germans.^13^ While these studies primarily compared the utilization of MR by Germans and foreign nationals, results from the representative health survey of the Robert Koch Institute also showed less frequent utilization for PMB, which is defined by a more differentiated approach (including place of birth or nationality at the time of birth), as compared to that for non-PMB.^14^ In contrast, Brzoska and colleagues found no difference in the use of MR between PMB, foreign nationals, and Germans in a study including individuals insured through the German Pension Insurance, which is the largest rehabilitation provider in Germany.^15^ A cross-sectional analysis of the first wave of the German Cohort Study on Work, Age, Health, and Work Participation in 2011, which included employed persons born in 1959 and 1965, reported no difference in overall use of MR between PMB (differentiated by first- and second-generation migrants) and non-PMB. However, first- generation migrants had a significantly lower probability of outpatient MR.^16^ In contrast, in a recent publication based on combined data from the first and second waves in 2014 of the previously mentioned cohort study, Breckenkamp and colleagues reported significantly higher odds of MR for first-generation migrants when controlling for various socioeconomic factors, such as education and income.^17^

These heterogeneous results may be partly due to different definitions of migration status. While some studies used survey data to assess migration background (MB), taking into account different indicators (such as place of birth and native language),^15^, ^16^, ^17^ other studies relied on routine data from the German Pension Insurance, which only reports on nationality.^12^ However, it can be assumed that equating MB and nationality only incompletely represents the population of PMB in Germany. Potential access barriers, such as information and language problems, might be more common among foreign nationals than among PMB, which includes many Germans with a migration history.^1^ This may account for the reported heterogeneity in the results on MR utilization. Whether and to what extent PMB differ from non-PMB in their use of MR has therefore not been conclusively clarified.^10^

Differences in health care utilization across a population can be explained by Andersen's behavioral model of health care utilization.^18^ The model distinguishes between predisposing factors, for example sociodemographic characteristics such as age, gender, and migration status, enabling factors such as a formal legal entitlement to MR and need factors, i.e. the need for MR due to a chronic disease.

The aim of the paper was to test whether there are differences between employees with and without a MB with regard to rehabilitation intention, application, and utilization, focusing on one of the most prevalent health disorders. The analysis is based on a sample of employees with back pain, as back pain is still the leading cause for utilization of MR in Germany. In addition, the study aimed to contribute to a better understanding of the relevance of the definition of MB. We therefore tested whether and how different definitions of MB were associated with rehabilitation intention, application, and utilization between PMB and non-PMB.

## Methods

### Study design and participants

We used data from a large cohort study that analyzed barriers to accessing rehabilitation and the effectiveness of MR for individuals with back pain.^19^^,^^20^ A random sample of 45,000 people was drawn from two pension agencies (German Pension Insurance North and German Pension Insurance Central Germany). In March 2017, baseline questionnaires were sent and linked to administrative data on rehabilitation measures in 2017 and 2018, if responders gave their consent. Employees aged 45 to 59 years who reported back pain at least once in the past three months were included. Exclusion criteria were people who had applied for or used MR during the previous four years, or who had ever applied for or received disability pension benefits.

Written informed consent on study aims, participation requirements and the right to refuse was obtained from all participants, and the trial conducted in accordance with the principles of the Declaration of Helsinki and Good Clinical Practice. The study was approved by the Ethics Committee of the University of Lübeck (15–144) and Martin Luther University Halle-Wittenberg (2015–49). The study was registered in the German Clinical Trials Register (DRKS00011554). The manuscript preparation considered the Strengthening the Reporting of Observational Studies in Epidemiology (STROBE) statement for cohort studies.^21^

### Outcomes

We distinguished three steps that must be completed in order to receive rehabilitation as outcomes of our analyses: intention to apply for MR, applying for MR, and utilizing MR. The intention to apply for MR was assessed via questionnaire by one binary variable (12): Do you intend to apply for rehabilitation within the next 12 months (no, yes)? Data on application for MR or use of MR were extracted from the administrative records of the two pension agencies and covered an observation period from study entry in 2017 until the end of 2018. Two binary variables indicated whether a rehabilitation measure was applied for or utilized.

### Migrant background

For our analyses, we used four different definitions of MB, which have previously been used in health research. The first definition followed the recommendations for epidemiological studies by Schenk and colleagues, and considered the country of birth of the parents, in addition to the country of birth of the person, and the persons native language.^22^ A differentiation is possible between non-migrants (non-PMB1), first-generation migrants (G1-PMB), and second generation migrants (G2-PMB). The second definition used the country of birth of the person and the parents. This definition differentiates between persons with no MB (non-PMB2), a one-sided MB (Uni-PMB), or a two-sided MB (Bil-PMB).^23^ In some cases, the nationality of the parents is also taken into account in this definition,^24^ but we omitted this since this information was not available in our data set. The third definition was based on language only and distinguished between persons whose native language is German (non-PMB3) and persons with another native language (Lg-PMB).^25^ Finally, as a fourth definition, the MB was derived from the nationality of an individual, as it is done in particular in the context of studies that rely on administrative data (non-PMB4 or Nat-PMB). All four definitions are shown in Table 1.

**Table 1:** Definitions for migrant background operationalization.

Definition	Acronym	Name	Condition	Source	Refs.
**1**	non-PMB1	no migrant background	Persons who were born in Germany and speak German, with both parents born in Germany	Questionnaire	^22^
	G1-PMB	Migrant background in first generation	Persons who were not born in Germany	Questionnaire	
	G2-PMB	Migrant background in second generation	Persons who were born in Germany with both parents not born in Germany	Questionnaire	
**2**	non-PMB2	no migrant background	Persons who were born in Germany with both parents born in Germany	Questionnaire	^23^
	Uni-PMB	One-sided migrant background	Persons who were born in Germany and with one parent not born in Germany	Questionnaire	
	Bil-PMB	Two-sided migrant background	Persons who were not born in Germany and with at least one parent not born in Germany or persons born in Germany with both parents not born in Germany	Questionnaire	
**3**	non-PMB3	Native German Speakers	Persons whose native language is German	Questionnaire	^25^
	Lg-PMB	Non-native Speakers	Persons whose native language is not German	Questionnaire	
**4**	non-PMB4	German nationality	Persons with German nationality	Administrative records	–
	Nat-PMB	non-German nationality	Persons without German nationality	Administrative records	

### Covariates

We assessed several covariates to include them in our models, considering potentially confounding factors in terms of the Health Care Utilization Model, such as age, gender, income, responsible pension agency, and health (i.e. need for rehabilitation). Age and sex were derived from the administrative records. All other covariates were assessed via questionnaire. Additional predisposing factors were partnership status (single vs. partnered), educational level (low vs. average vs. high), net household income (under 1500 Euros vs. 1500 to 3000 Euros vs. over 3000 Euros), job position (blue-collar vs. white-collar), knowledge of rehabilitation application procedures (two dichotomous items summed up), negative family-related outcome expectations (two dichotomous items summed up), and negative work-related outcome expectations (two dichotomous items summed up). We considered social support for rehabilitation application from family and friends (three dichotomous items summed up), social support for rehabilitation application from physicians and therapists (three dichotomous items summed up), and responsible pension agency as enabling factors. Further variables assessed the health status to address potential need factors. We assessed height and weight to calculate the body mass index. We considered the four-level pain grading by von Korff and colleagues^26^ derived from three variables of the Chronic Pain Questionnaire (i.e., pain disability, pain intensity, and days of disability). Pain grade I represents low intensity pain associated with limited disability, grade II represents high intensity pain and limited disability, grade III represents moderate disabling pain regardless of pain intensity, and grade IV represents severely disabling pain regardless of pain intensity.^26^ Furthermore, we assessed depressive symptoms using the eight-item depression module of the Patient Health Questionnaire (PHQ-8).^27^

### Statistical analysis

Descriptive statistics including absolute and relative frequencies and means with standard deviations characterized the full sample, and samples stratified on the different definitions of migration status. To determine the associations of MB and the intention to apply for MR, logistic regression models were calculated according to the different migrant status definitions. We added age, gender, partnership, education, income, job position, health-related variables, and variables on support factors and barriers for the rehabilitation request as covariates to the model for adjustment. We report adjusted predicted probabilities.^28^

Time at risk for application and utilization of MR was computed from the date of receipt of the questionnaire. Observations were censored at the end of 2018. Proportional hazard models were fitted to determine the association of different definitions of migration status when considering relevant covariates. Hazard ratios (HRs) and corresponding 95% confidence intervals (CIs) were estimated. Adjustment was performed using the approach as described above. In the logistic regression and hazard models pension agency was considered as a fixed effect.

Missing values on the variables collected by questionnaire ranged from 0·5% (educational level) to 2·8% (PHQ-8). The missing data analysis procedures used missing at random assumptions. We tested this assumption by predicting the missingness of values based on our baseline variables in logistic regression models, and identified auxiliary variables that are associated with missingness,^29^ e.g. educational level was significantly associated with the missingness of net household income (odds ratio = 0·48; *p* < 0·001). This suggests that educational level is a potential predictor of missingness which makes the missing at random assumptions more plausible. Missing self-reported baseline data were imputed using chained equations.^30^ Independent variables without missing values (age, sex, migrant status variables, and outcomes as recommended by Kontopantelis and colleagues)^31^ were included as covariates in the imputation model. We created 20 independent data sets with complete values. Parameter estimates of the proportional hazard and logistic regression models were combined in accordance with Rubin's rules.^32^ We additionally performed a complete-case analysis for the outcome utilization of MR.

Application of MR is a prerequisite for the utilization. Applications for MR may be rejected for medical reasons or due to incomplete documents. In addition, it is possible that insured persons did not commence approved services. Therefore, in a subsample in which only applicants were analyzed, we tested (with a logistic regression model) the association of MB definitions when considering the granting rate of MR and the adjustment described above.

The statistical test results were regarded as significant if the two-sided p-value of a test was less than 0·05. All calculations were performed in Stata SE 16.

### Role of funding

The study was funded by the German Research Foundation (grant numbers: BE 5885/2–1; MA 6981/2–1). The funding source did not have any involvement in study design, data collection and management, data analysis or interpretation, preparation, review, or approval of the manuscript, or the decision to submit the manuscript for publication. DF and HB had access to the data and took the decision to submit for publication.

## Results

### Recruitment and participants

**Figure 1:**
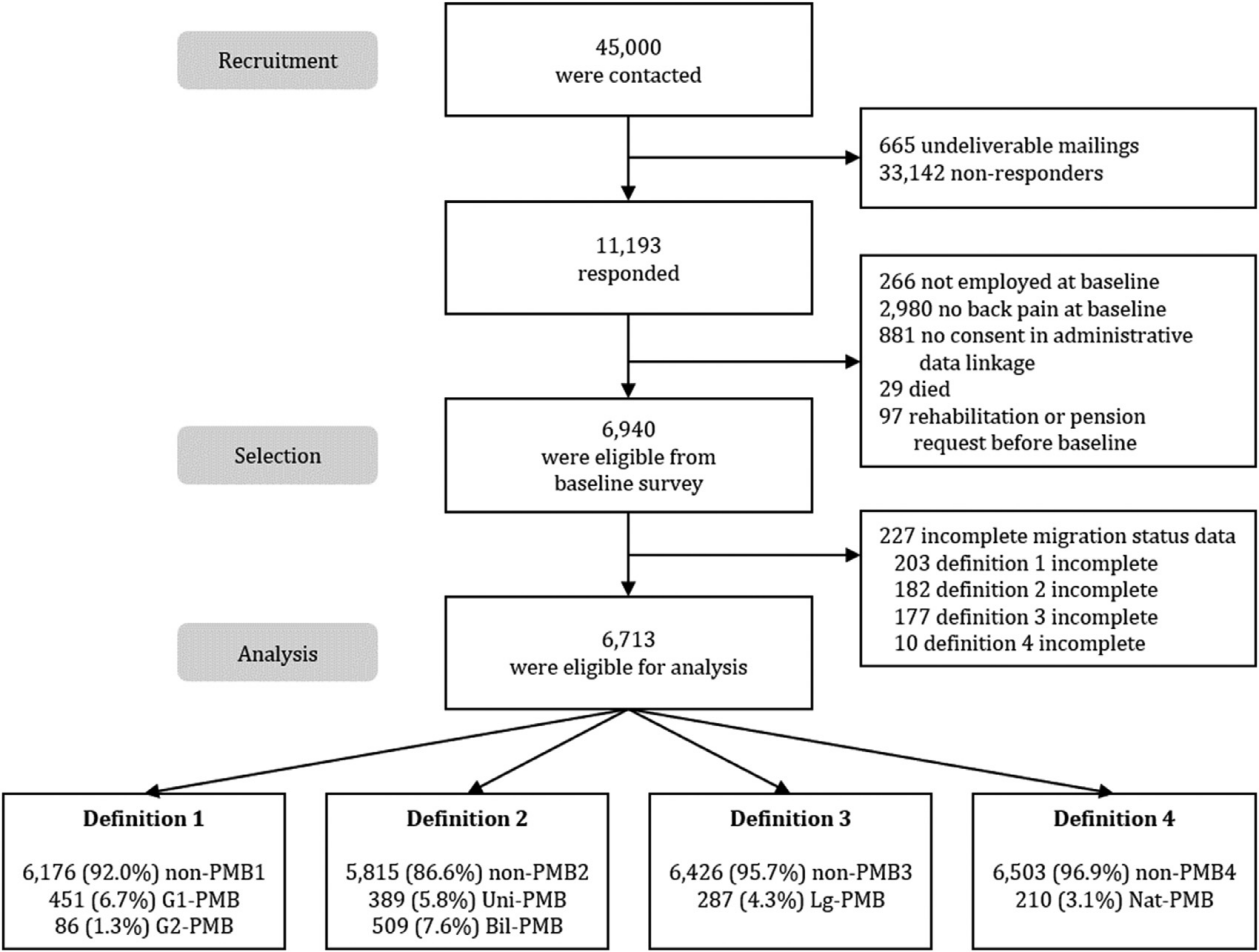
Flow of participants and distribution of definitions of migration background.

A total of 45,000 persons were contacted via postal questionnaires. A total of 11,193 persons completed the baseline questionnaire between March 2017 and August 2017. We excluded 881 persons due to the lack of consent to the linkage of the questionnaire and administrative data or non-availability of their administrative data. Another 266 persons were excluded since they were not employed. In all, 29 persons died. A total of 2,980 persons did not report back pain at baseline. An additional 97 persons were excluded, as they applied for a rehabilitation measure or a disability pension before the receipt of the baseline questionnaire. Finally, 227 persons were excluded due to missing data on MB variables. Missing data on various migration status definitions were 2·9%, 2·6%, 2·6%, and 0·1% of 6,940 participants for definition 1, 2, 3, and 4, respectively. A total of 6,713 individuals were considered for analysis (Figure 1). The mean age was 52·3 years (standard deviation = 4·1), and 57·7% were women. About 85·1% of the respondents were in a relationship, 46·4% were blue-collar workers. The two pension agencies (German Pension Insurance North and German Pension Insurance Central Germany) were represented in comparable proportions (Central Germany: *n* = 3,366; North: *n* = 3,347; not shown) Table 2. presents selected sample characteristics and outcomes stratified for definitions of MB.

**Table 2:** Selected sample characteristics stratified by different definitions of migrant background.

	Definition 1			Definition 2			Definition 3		Definition 4	
	non-PMB1 (*n* = 6,176)	G1-PMB (*n* = 451)	G2-PMB (*n* = 86)	non-PMB2 (*n* = 5,815)	Uni-PMB (*n* = 389)	Bil-PMB (*n* = 509)	non-PMB3 (*n* = 6,426)	Lg-PMB (*n* = 287)	non-PMB4 (*n* = 6503)	Nat-PMB (*n* = 210)
*Sociodemographic*										
Sex: female, n (%)	3,553 (57·3)	265 (58·8)	56 (65·1)	3,328 (57·2)	240 (61·7)	306 (60·2)	3,707 (57·7)	167 (58·1)	3,762 (57·9)	112 (53·3)
Age, M (SD)	52·3 (4·1)	52·0 (4·1)	53·4 (3·8)	52·3 (4·1)	52·5 (4·1)	52·2 (4·1)	52·3 (4·1)	51·6 (4·0)	52·3 (4·1)	51·4 (4·1)
Partnership: yes, n (%)	5,168 (85·0)	384 (87·5)	72 (83·7)	4,852 (84·8)	342 (88·6)	430 (86·4)	5,380 (85·1)	244 (86·2)	5,445 (85·1)	179 (86·9)
Educational level, n (%)										
Low	1,071 (17·4)	139 (31·4)	14 (16·3)	1,016 (17·5)	63 (16·2)	145 (28·9)	1,130 (17·7)	94 (33·7)	1,146 (17·7)	78 (38·1)
Medium	4,560 (74·1)	222 (50·1)	67 (77·9)	4,282 (73·9)	294 (75·6)	273 (54·5)	4,714 (73·6)	135 (48·4)	4,767 (73·6)	82 (40·0)
High	523 (8·5)	82 (18·5)	5 (5·8)	495 (8·5)	32 (8·2)	83 (16·6)	560 (8·7)	50 (17·9)	565 (8·7)	45 (22·0)
Net income, n (%)										
<1500 €	796 (13·1)	102 (23·3)	17 (20·0)	751 (13·2)	51 (13·5)	113 (22·7)	837 (13·3)	78 (27·9)	865 (13·6)	50 (24·4)
1500 - 3000 €	3,535 (58·3)	238 (54·3)	51 (60·0)	3,313 (58·0)	234 (61·9)	277 (55·7)	3,674 (58·3)	150 (53·6)	3,715 (58·2)	109 (53·2)
>3000 €	1,733 (28·5)	98 (22·4)	17 (20·0)	1,648 (28·9)	93 (24·6)	107 (21·5)	1,796 (28·5)	52 (18·6)	1,802 (28·2)	46 (22·4)
Labor: blue-collar, n (%)	2,831 (46·1)	218 (49·4)	46 (54·1)	2,660 (46·0)	181 (46·7)	254 (51·0)	2,955 (46·3)	140 (50·2)	2,997 (46·4)	98 (48·0)
*Outcomes*										
Intention: yes, n (%)	764 (12·5)	71 (16·1)	13 (15·5)	728 (12·7)	38 (9·8)	82 (16·4)	807 (12·7)	41 (14·6)	829 (12·9)	19 (9·2)
Application: yes, n (%)	580 (9·4)	24 (5·3)	6 (7·0)	543 (9·3)	38 (9·8)	29 (5·7)	595 (9·2)	15 (5·2)	604 (9·3)	6 (2·9)
Utilization: yes, n (%)	489 (7·9)	21 (4·7)	4 (4·7)	458 (7·9)	32 (8·2)	24 (4·7)	501 (7·8)	13 (4·5)	509 (7·8)	5 (2·4)
*Health*										
Pain grading, n (%)										
Grade I or II	4,866 (78·8)	300 (66·5)	60 (69·8)	4,576 (78·7)	309 (79·4)	341 (67·0)	5,032 (78·3)	194 (67·6)	5,079 (78·1)	147 (70·0)
Grade III or IV	1,310 (21·2)	151 (33·5)	26 (30·2)	1,239 (21·3)	80 (20·6)	168 (33·0)	1,394 (21·7)	93 (32·4)	1,424 (21·9)	63 (30·0)
Depression, M (SD)	5·9 (4·5)	7·2 (5·1)	6·4 (4·4)	5·92 (4·5)	6·26 (4·3)	7·1 (5·0)	6·0 (4·4)	7·2 (5·0)	6·0 (4·5)	6·7 (4·8)

*Note: M* = mean; SD = standard deviation; valid percentages were reported.

### Migration background

The different definitions of MB showed different proportions of PMB in our sample (Table 2). Based on definition 1, a proportion of 8% (G1-PMB: 6·7%; G2-PMB: 1·3%) of PMB was observed. Definition 2 showed a proportion of 13·4% (Uni-PMB: 5·8%; Bil-PMB: 7·6%) of PMB. Regarding definition 3, we observed a proportion of 4·3% (*n* = 287) whose native language is not German (Lg-PMB). Lastly, definition 4 indicated that a proportion of 3·1% (*n* = 210) had a nationality other than German (Nat-PMB). Different characteristics with regard to education, income, and health were observed depending on the various definitions of migrant status (Table 2).

With regard to the overlap of definitions, it can be seen that 5·8% of the sample have no MB according to definition 1, who would be classified as having a one-sided MB according to definition 2. Defining MB by language would classify 3·7% of the sample as having no MB, who would be first- and second-generation migrants according to definition 1. In the definition by nationality, 4·9% have no MB, who would be first- and second-generation migrants according to definition 1 (Online Supplement 1).

### Intention to apply for, applying for and utilization of medical rehabilitation services

Absolute risks of an intention to apply for MR differed regarding the various definitions of MB (Table 2). Time at risk for rehabilitation events considered a maximum follow-up time of 21 months. In total, the proportion of people who applied for and utilized MR were 9·1% (*n* = 610) and 7·7% (*n* = 514), respectively (Table 2). Over half of all rehabilitation measures (56·6%) were granted due to diseases of the musculoskeletal system (M00-M99; International Statistical Classification of Diseases and Related Health Problems, ICD-10) (not shown). Proportion of application and utilization differed between various definitions. People with a one-sided MB showed the highest proportion of rehabilitation application and utilization. They were comparable to Non-PMB according to all definitions. All other subgroups of PMB showed clear underutilization of rehabilitation measures (about a half) when compared to that of non-PMB. The lowest proportion (rehabilitation application: 2·9%) was seen in people without German nationality (Table 2).

### Multivariate associations of migration and rehabilitation outcomes

Results of the logistic regression and proportional hazard models are shown in Table 3. An intention to apply for MR was not significantly associated with different MB definitions. After adjusting for sociodemographic variables, health characteristics, and potential support factors and barriers to applying for rehabilitation, significant associations of migrant status definitions and rehabilitation application, and utilization were observed. In the adjusted model, the risk of application and utilization was halved in people with G1-PMB according to definition 1 when compared to non-PMB1 (application: HR = 0·47; 95% CI 0·31; 0·71; utilization: HR = 0·46; 95% CI 0·29; 0·72). Similarly, people with a two-sided MB (Bil-PMB) according to definition 2 had half the risk of rehabilitation application (HR = 0·50; 95% CI 0·34; 0·73) and utilization (HR = 0·47; 95% CI 0·31; 0·72) compared to non-PMB2. Using definition 3, the risk ratio estimate was again very similar. The risk of people whose native language was not German was decreased by 47% compared to non-PMB3 (application: HR = 0·53; 95% CI 0·32; 0·90; utilization: HR = 0·52; 95% CI 0·30; 0·91). The strongest association of migration status and rehabilitation use was seen when considering definition 4. The risk of application for and utilization of MR was reduced by about 70% in Nat-PMB compared to non-PMB4 (application: HR = 0·31; 95% CI 0·14; 0·70; utilization: HR = 0·29; 95% CI 0·12; 0·72).

**Table 3:** Associations of intention to apply for, application for, and utilizing of medical rehabilitation and migrant status variations.

	Intention			Application			Utilization		
	OR	95% CI	p	HR	95% CI	p	HR	95% CI	p
**Definition 1**									
non-PMB1	Ref.			Ref.			Ref.		
G1-PMB	1·34	0·96; 1·86	0·084	0·47	0·31; 0·71	**< 0·001**	0·46	0·29; 0·72	**0·001**
G2-PMB	1·02	0·51; 2·06	0·946	0·61	0·27; 1·38	0·236	0·50	0·18; 1·33	0·163
**Definition 2**									
non-PMB2	Ref.			Ref.			Ref.		
Uni-PMB	0·70	0·47; 1·06	0·092	0·98	0·70; 1·36	0·891	0·97	0·69; 1·41	0·862
Bil-PMB	1·30	0·95; 1·77	0·098	0·50	0·34; 0·73	**< 0·001**	0·47	0·31; 0·72	**< 0·001**
**Definition 3**									
non-PMB3	Ref.			Ref.			Ref.		
Lg-PMB	1·35	0·90; 2·03	0·150	0·53	0·32; 0·90	**0·019**	0·52	0·30; 0·91	**0·023**
**Definition 4**									
non-PMB4	Ref.			Ref.			Ref.		
Nat-PMB	0·81	0·47; 1·41	0·460	0·31	0·14; 0·70	**0·005**	0·29	0·12; 0·72	**0·007**

*Note: n* = 6,713; OR = odds ratio; HR = hazard ratio; CI = confidence interval; Estimates were calculated using imputed data from 20 data sets, and were fully adjusted for sociodemographic characteristics, socioeconomic characteristics, health characteristics, and potential supporting factors and barriers.

A complete-case analysis (*n* = 5,172) showed comparable results of the model estimates (not shown).

### Additional analysis with rehabilitation applicants

In a subsample with applicants only (*n* = 610), we analyzed the association between definitions of MB and rehabilitation utilization. We found no statistically significant differences of utilization regarding different MB definitions (not shown).

## Discussion

Despite increased efforts in recent years, comparatively little data is available on health care, particularly rehabilitative care, of PMB in Germany. As stated in the WHO's "Framework of Priorities and Guiding Principles to Promote the Health of Refugees and Migrants", societal efforts should be directed towards the goal of reducing health inequalities in the population and guaranteeing the best possible physical and mental health for all people.^33^ Therefore, it is necessary to identify potential inequalities and barriers to rehabilitation, and to identify needs and opportunities for action by institutions and policy makers.

This study showed that employed people reporting back pain with a MB had a significantly reduced risk for an application and utilization of MR, even after adjusting for relevant predisposing, enabling, and need factors according to the Andersen behavioral model of health care utilization. Four key findings can be derived from the results.

First, we could demonstrate that participants with MB had a lower unconditional probability of applying for and utilizing MR. This was the case for all definitions except for persons with a one-sided MB, although the probability for rehabilitation application or utilization varied for the different definitions of migration status. Moreover, the results indicate that underutilization of MR by persons with a MB is not solely due to differences in socioeconomic status and health. The adjustment made for barriers and facilitators to utilization suggests that differences in knowledge about application, social support from family and friends or physicians and therapists, and negative outcome expectations cannot conclusively explain the difference in utilization. However, it is likely that the covariates we used only partially captured potential support factors and barriers. Our finding of an underutilization by PMB is in line with studies predominantly based on administrative data that reported lower utilization of PMB.^12^^,^^13^ In contrast, however, more recent findings indicated that individuals with a MB have a similar or even greater likelihood of using MR.^16^^,^^17^ We therefore suggest that future studies should attempt to identify further factors that could explain differences in the utilization of MR by PMB. For this purpose, studies with a larger number of cases would be desirable, which would allow a differentiation of different migration parameters (e.g., country of origin, duration of stay).

Second, with regard to the different definitions, our results suggest that studies on the application and utilization of MR may overestimate the influence of MB if it is represented by the nationality of individuals only, as is often the case in the analysis of administrative data.^10^^,^^12^ Although PMB had a lower likelihood of applying regardless of definition, this association was most pronounced for individuals with foreign nationality compared to people with German nationality.

Third, persons with a one-sided MB (definition 2) did not differ in utilization from persons without a MB. This implies that studies using a broad definition of MB (e.g., all individuals who have at least one parent who was not born in Germany) may underestimate the association between MB and utilization compared with narrower definitions. Therefore, further research should also address the question of which factors within the migrant population cause the lower utilization as well as which characteristics or circumstances can be seen as enabling factors according to the Andersen model of healthcare utilization. The overlap of Nat-PMB, G1-PMB, and Bil-PMB, and the lower uptake in these groups is possibly an indication that the duration of residence in Germany could be one of these factors, as a shorter duration of stay can be assumed for these groups based on the definitions. In a study from Norway, for example, a correlation between length of stay and utilization of primary care was reported for migrants.^34^

Fourth, in our sensitivity analysis of the subsample of applicants, we found no statistically significant association between migration status and utilization of the requested MR, regardless of which of the four definitions we used. Accordingly, there are no differences in the conditional probability of utilization after having applied between PMB and non-PMB. Therefore, differences in utilization can be explained by differences in application. Moreover, we found no difference in intention to apply for MR between persons with and without a MB, regardless of which operationalization of MB we used. Therefore, the barriers of application may lie between personal consideration of MR and concrete application. Based on focus group interviews with PMB, Schwarz and colleagues describe a set of barriers that can make it difficult for people with a MB to use MR.^35^ In addition to person-related barriers, such as a lack of German language skills and lack of or incorrect knowledge about rehabilitation, they also cite system-related barriers, such as a lack of intercultural openness of institutions (e.g., in terms of dietary, prayer, and gender-specific regulations) as a key barrier. Such system-related barriers could possibly evoke a turning away from the original intention of utilization if affected persons gain the impression that culturally specific needs are only insufficiently considered during the clinic stay. The observed difference between intention and consecutive application and utilization may imply that future strategies to integrate PMB into existing health care should not focus solely on publicizing existing services but should consider targeted support (e.g., assistance with formal application, or contacts for migrant-specific concerns regarding utilization).

The results must be interpreted in light of the following limitations. Firstly, the response rate of only approximately one-quarter was low, although this is common in postal surveys. There might be unobserved differences between responders and non-responders. Selection bias due to selective non-participation might have biased the estimates. Secondly, the cohort study considered participants aged 45 to 59 years and reported back pain; therefore, representativeness is limited. Younger generations with a different migration history (e.g., a different history in the country of origin and different motives for migration) might differ from our sample both in terms of their sociodemographic and socioeconomic backgrounds and in their utilization, and the proportion of migration status subgroups (e.g., first-generation vs. second-generation) will probably differ. Furthermore, our data only allowed us to consider whether individuals or their parents were born in another country, but not in which country they were born. However, country of origin could also be a factor with regard to utilization. Thirdly, the baseline survey was conducted in German. This may have led to lower study participation by people with a MB due to language problems. Overall, the proportion of people with non-German citizenship was around 17% among all persons insured by the German Pension Insurance. Fourthly, the size of the groups and the number of outcome events within the different groups of PMB included in the proportional hazard models were very low compared to the events of non-PMB used as reference group (e.g., four events in G2-PMB compared to 489 events in non-PMB1 for utilization of MR). This should be considered when interpreting the results.

These limitations are balanced by several strengths. Firstly, the large sample was randomly drawn from the register of the German Pension Insurance. The sample was restricted to persons aged 45 to 59 years, who are the primary consumers of MR. Furthermore, we included persons with self-reported back pain (i.e., a group with a biopsychosocial health problem and particularly at risk of early retirement and permanent work disability). Secondly, the linkage of questionnaire and administrative data records allowed a follow-up without sample attrition. Thirdly, the use of administrative data ensured a valid and reliable assessment of the study outcomes. Thus, recall bias and misclassification of rehabilitation application and utilization were avoided.

In summary, although generalization of the results is limited to the specific population of our study (i.e., employed persons aged 45 to 59 years with self-reported back pain), it complements the current evidence indicating an underutilization of MR in PMB, except for people with a one-sided MB. Our results revealed that the different definitions of MB are differently sensitive to identify PMB. Definitions 1 (first- and second-generation) and 2 (one-sided and two-sided) led to the highest proportions of PMB in our sample. The higher the proportion was, the greater the heterogeneity of the included individuals. In this case, differentiation of PMB seems to be valuable, and we suggest that migration status should not be treated as a dichotomous characteristic (PMB or non-PMB). We recognized that people with a one-sided MB had very similar outcomes to non-migrants, indicating no obvious disparities in health and health care utilization. This differentiation may have only minor impact in health service research when looking at disparities. Differing between first- and second-generation migrants is more meaningful in our view. Both groups were less likely to use MR, but differed regarding sociodemographic characteristics (e.g., educational level and net income), which may be associated with burden of disease and health care utilization.^36^ Therefore, we recommend a differentiation of first- and second-generation migrants for health service research.

### Funding

The study was funded by the German Research Foundation (grant numbers: BE 5885/2–1; MA 6981/2–1). The German Research Foundation functions as a self-governing institution for the promotion of science and research in Germany. We acknowledge financial support by Land Schleswig- Holstein within the funding programme Open Access Publikationsfonds.

### Contributors

DF and HB had full access to all of the data in the study and take responsibility for the integrity of the data and the accuracy of the data analysis. The authors have contributed to the work as follows: DF (conceptualization, methodology, data curation, formal analysis, validation, investigation, visualization and writing – original draft); HB (conceptualization, methodology, formal analysis, validation, visualization and writing – original draft); JMZ (data curation, investigation and writing – review and editing); NS (data curation, investigation and writing – review and editing); AG (data curation, investigation and writing – review and editing); WM (project administration, funding acquisition, supervision and writing – review and editing); and MB (project administration, funding acquisition, supervision and writing – review and editing).

### Data sharing statement

The datasets generated for this study are available on request to the corresponding author.

## Declaration of interests

Prof. Bethge and Prof. Mau report grants from German Research Foundation, during the conduct of the study. The remaining authors have nothing to disclose.

## Appendix Supplementary materials

Image, application 1
