# Supplementary material for: Rehabilitation utilization of non-migrant and migrant persons with back pain: A cohort study using different definitions of migrant background
Source: eClinicalMedicine. 2022 Mar 21;46:101351. doi: 10.1016/j.eclinm.2022.101351 (PMC8938875; doi:10.1016/j.eclinm.2022.101351)
Supplement: Supplementary file 1 [file mmc1.docx]

Online Supplement 1: Overlap of the operationalizations of migrant status in the sample.

|  | **Definition 1** | | |  | **Definition 2** | | |  | **Definition 3** | |  |
| --- | --- | --- | --- | --- | --- | --- | --- | --- | --- | --- | --- |
|  | non-PMB1  [n (%)] | G1-PMB  [n (%)] | G2-PMB  [n (%)] | Total  [n (%)] | non-PMB2  [n (%)] | Uni-PMB  [n (%)] | Bil-PMB  [n (%)] | Total  [n (%)] | non-PMB3  [n (%)] | Lg-PMB  [n (%)] | Total  [n (%)] |
| **Definition 2** |  |  |  |  |  |  |  |  |  |  |  |
| non-PMB2 | 5,787 (86·2) | 28  (0·4) | 0  (0·0) | 5,815 (86·6) |  |  |  |  |  |  |  |
| Uni-PMB | 389  (5·8) | 0  (0·0) | 0  (0·0) | 389  (5·8) |  |  |  |  |  |  |  |
| Bil-PMB | 0  (0·0) | 423  (6·3) | 86  (1·3) | 509  (7·6) |  |  |  |  |  |  |  |
| Total | 6,176 (92·0) | 451  (6·7) | 86  (1·3) | 6,713 (100·0) |  |  |  |  |  |  |  |
| **Definition 3** |  |  |  |  |  |  |  |  |  |  |  |
| non-PMB3 | 6,176 (92·0) | 164  (2·4) | 86  (1·3) | 6,426 (95·7) | 5,815  (86·6) | 389  (5·8) | 222  (3·3) | 6,426  (95·7) |  |  |  |
| Lg-PMB | 0  (0·0) | 287  (4·3) | 0  (0·0) | 287  (4·3) | 0  (0·0) | 0  (0·0) | 287  (4·3) | 287  (4·3) |  |  |  |
| Total | 6,176 (92·0) | 451  (6·7) | 86  (1·3) | 6,713 (100·0) | 5,815  (86·6) | 389  (5·8) | 509  (7·6) | 6,713 (100·0) |  |  |  |
| **Definition 4** |  |  |  |  |  |  |  |  |  |  |  |
| non-PMB4 | 6,173 (92·0) | 247  (3·7) | 83  (1·2) | 6,503 (96·9) | 5,815  (86·6) | 386  (5·8) | 302  (4·5) | 6,503  (96·9) | 6,382  (95·1) | 121  (1·8) | 6,503  (96·9) |
| Nat-PMB | 3  (0·0) | 204  (3·0) | 3  (0·0) | 210  (3·1) | 0  (0·0) | 3  (0·0) | 207  (3·1) | 210  (3·1) | 44  (0·7) | 166  (2·5) | 210  (3·1) |
| Total | 6,176 (92·0) | 451  (6·7) | 86  (1·3) | 6,713 (100·0) | 5,815  (86·6) | 389  (5·8) | 509  (7·6) | 6,713  (100·0) | 6,426  (95·7) | 287  (4·3) | 6,713  (100·0) |

*Note:* The percentages are given in comparison to the total number of cases (n = 6,713); deviations due to rounding.
